# Nutlin-3 inhibits androgen receptor-driven c-FLIP expression, resulting in apoptosis of prostate cancer cells

**DOI:** 10.18632/oncotarget.12542

**Published:** 2016-10-09

**Authors:** Ian R. Logan, Urszula L. McClurg, Dominic L. Jones, Daniel J. O'Neill, Fadhel S. Shaheen, John Lunec, Luke Gaughan, Craig N. Robson

**Affiliations:** ^1^ Institute of Cellular Medicine, Newcastle University, Newcastle upon Tyne, Tyne and Wear, United Kingdom

**Keywords:** prostate cancer, MDM2, androgen receptor, c-FLIP, anti-androgens

## Abstract

Inhibition of androgen receptor (AR) signalling represents the conventional medical management of prostate cancer. Ultimately this treatment fails because tumors develop an incurable, castrate resistant phenotype, resulting in an unmet need for new treatments in prostate cancer. The AR remains a viable therapeutic target in castrate resistant disease, such that novel ways of downregulating AR activities are attractive as potential treatments. Here we describe a mechanism by which the AR can be downregulated by the MDM2 antagonist Nutlin-3, resulting in loss of pro-survival c-FLIP gene expression and apoptosis. We additionally show that loss of c-FLIP sensitises prostate cancer cells to Nutlin-3. Finally, we demonstrate that the unrelated MDM2 antagonist Mi-63 also impinges upon AR signalling, supporting the concept of future treatment of prostate cancer with MDM2 antagonists.

## INTRODUCTION

Numerous lines of evidence implicate the androgen receptor (AR) in all stages of prostate cancer, the most common male malignancy. Pharmacological inhibition of AR activity by androgen deprivation therapy (ADT) is the mainstay of treatment in prostate cancer, where curative surgery is contraindicated [1]. The major caveat to ADT is the development of castrate resistant prostate cancer (CRPC), a lethal form of the disease, which emerges after long-term administration of anti-androgen compounds [2, 3]. Nevertheless, AR activity is retained in CRPC, highlighting the concept that alternative means of targeting AR could find clinical utility [4–7].

Evasion of apoptosis is widely accepted to be important in tumorogenesis and chemotherapeutic resistance. The pro-survival FLICE-inhibitory protein (c-FLIP) is a master regulator of apoptosis that blocks activation of the extrinsic (death receptor-mediated) apoptotic pathway by inhibiting the formation of the death-inducing signalling complex (DISC) and subsequent inactivation of PROCASPASE 8 [8]. c-FLIP is aberrantly expressed in both high grade prostate cancer and CRPC and is a direct target for AR-mediated transcriptional activity [9–11]; the c-FLIP gene promoter contains defined androgen response elements (AREs). This makes c-FLIP the most extensively studied androgen-responsive pro-survival gene and it has been proposed that c-FLIP mediated survival is partly responsible for the development of CRPC. A means to block c-FLIP activity or expression is therefore potentially beneficial.

p53, the most commonly mutated tumor suppressor gene in human cancers, encodes a transcription factor responsible for cell cycle arrest and apoptosis in response to DNA damage and chemotherapeutic drugs [12]. Interestingly, localised prostate tumors contain relatively few p53 mutations, whereas metastatic tumors harbor far more mutations [13, 14]. This suggests that the less aggressive stages of the disease retain functional p53. The Mdm2 gene, encoding an E3 ubiquitin ligase enzyme that is a key negative regulator of p53, is amplified in around 7% of all tumors as an alternative means of inactivating p53, although this rarely occurs in prostate cancer [15]. These observations raise the question of enhancing intact p53 activity as a means of treatment in prostate cancer, at least in the early stages of disease. Such an approach might allow either postponement of traditional anti-androgen treatment and the onset of CRPC, or simultaneous / cycling administration of p53 activating agents and anti-androgens, to more effectively eliminate cancerous cells whilst they remain vulnerable to both strategies. Intensive research into restoring p53 activity in tumors has produced small molecule inhibitors of the p53-MDM2 interaction, including the Nutlin compounds and their derivatives, some of which are undergoing clinical evaluation [16]. Nutlin-3 is the most characterized of these agents and the first to be reported as having anti-tumor activity *in vivo*, including inhibition of LNCaP prostate cancer xenograft growth [17, 18]. Interestingly, the LNCaP prostate cancer cell line, which expresses functional AR exhibited distinct apoptotic sensitivity to Nutlin-3, an observation that is thus far unexplained. MDM2 gene amplification exists in other cell lines that are highly sensitive to Nutlin-3, but not in LNCaP cells [18]. AR itself has previously been shown to be a target for MDM2-mediated ubiquitination and degradation [19, 20]. Although Nutlin-3 disrupts the p53-MDM2 interaction, the MDM2 E3 ubiquitin ligase activity for non-p53 targets is preserved in the presence of Nutlin-3 [21], raising the hitherto unexplored possibility that AR could be downregulated by MDM2, in the presence of Nutlin-3. This might account for the notable sensitivity of LNCaP cells to Nutlin-3.

Previous work has indeed demonstrated that Nutlin-3 treatment reduces AR protein levels and produces cell cycle arrest and apoptosis in prostate cancer cells expressing active AR and wild-type p53 [22, 23]. Additionally, Nutlin-3 also boosts the anti-tumor effect of castration in mice [23], however the relative importance of Nutlin-3 on AR-mediated survival versus p53-mediated cell cycle arrest and apoptosis remains unexplored. Other important questions remain around Nutlin-3 activity in prostate cancer, including the mechanism of AR downregulation, the relative contribution of cell cycle arrest versus apoptosis, and the impact upon AR-driven pro-survival genes. Moreover, the effects of Nutlin-3 combined with conventional direct AR antagonists *in vivo* is unclear, as is the question of whether newer anti-androgens such as enzalutamide (MDV3100) [24], which affords increased patient survival in CRPC [25], might also be useful in combination with agents such as Nutlin-3.

Here we address some of these questions by providing new insight into Nutlin-3 activity in prostate cancer cells. We show that sensitivity to Nutlin-3 treatment correlates with AR dependency in different cells models, that otherwise have the same p53 response. This suggests that AR signalling is an important determinant of Nutlin-3 efficacy, beyond the p53 response, and offers an explanation for the marked sensitivity of LNCaP cells to Nutlin-3. We go on to show that Nutlin-3 treatment increases AR-MDM2 interactions resulting in reduced AR levels, loss of AR from the pro-survival c-Flip gene promoter, downregulation of c-FLIP expression and subsequent downstream cleavage of pro-apoptotic CASPASE-8. Consequently, Nutlin-3 combined with anti-androgen treatments, or AR depletion, results in widespread apoptosis. Conversely, Nutlin-3 combined with anti-androgen treatment did not enhance cell cycle arrest beyond that observed with Nutlin-3 alone, implying that apoptosis is the key mechanism at play. We propose that prostate cancers retaining AR and p53 signalling might have special significance in the clinical application of MDM2 inhibitors in order to prevent or delay the development of CRPC, which inevitability emergences with the conventional use of anti-androgens.

## RESULTS

### AR dependency correlates with sensitivity to Nutlin-3 in prostate cancer cell lines

**Figure 1:**
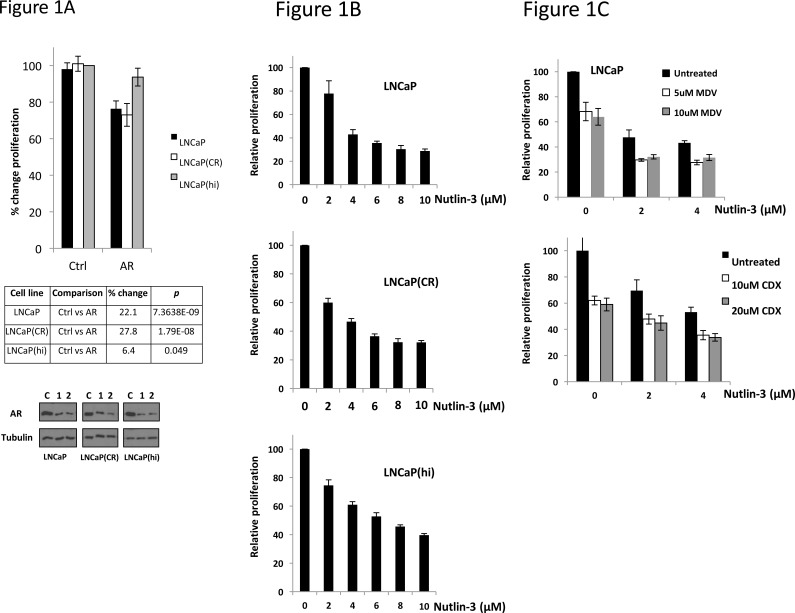
Androgen dependency correlates with sensitivity to Nutlin-3 **A.** Cell lines indicated were reverse transfected in 96 well plates at a density of 10,000 per well (*n* = 8) with control or AR siRNA 1 then subject to WST-1 proliferation assay 72 hr later. Immunoblotting shows level of AR knockdown between cells lines with two different AR siRNA sequences (C, control siRNA, 1 AR siRNA, 2 AR siRNA). **B.** Indicated cell lines were treated with Nutlin-3 in 96 well plates then subject to WST-1 proliferation assay 72 hr later. **C.** LNCaP cells were treated with combinations of MDV3100 (MDV) or Casodex (CDX) and Nutlin-3 in 96 well plates, then subject to WST-1 proliferation assay 72 hr later. Data are representative of a single experiment, error bars ±SD.

To determine whether any functional link might exist between AR signalling and the p53-MDM2 interaction, we first examined the sensitivity of 3 related prostate cancer cell lines, with differing dependency on AR, to Nutlin-3. As shown in Figure 1A, siRNA-mediated depletion of AR produced a reduction in proliferation to differing extents 72 hr post-transfection; low passage number parental LNCaP and a casodex-resistant variant LNCaP(CR) demonstrated modest, approximately 25% reduction in proliferation upon AR silencing. Higher passage number cells, LNCaP(hi), however were significantly less dependent upon AR for their proliferation, despite similar levels of AR knockdown to the other cells, as shown by immunoblotting. We next applied increasing doses of Nutlin-3 onto the three cell types (Figure 1B) in proliferation assays. Whereas the concentration of Nutlin-3 required to produce a decrease in proliferation by 50% (IC_50_) was approximately 3μM for both LNCaP and LNCaP(CR) cells, the less AR-dependent LNCaP(hi) cells exhibited an IC_50_ of 6μM Nutlin-3. Finally, we treated LNCaP cells with the direct AR antagonists enzalutamide or casodex in combination with Nutlin-3 for 72 hr (Figure 1C) before measuring proliferation. Both AR antagonists sensitized LNCaP cells to Nutlin-3. Overall, these data demonstrate that AR activity correlates with sensitivity to Nutlin-3.

**Figure 2:**
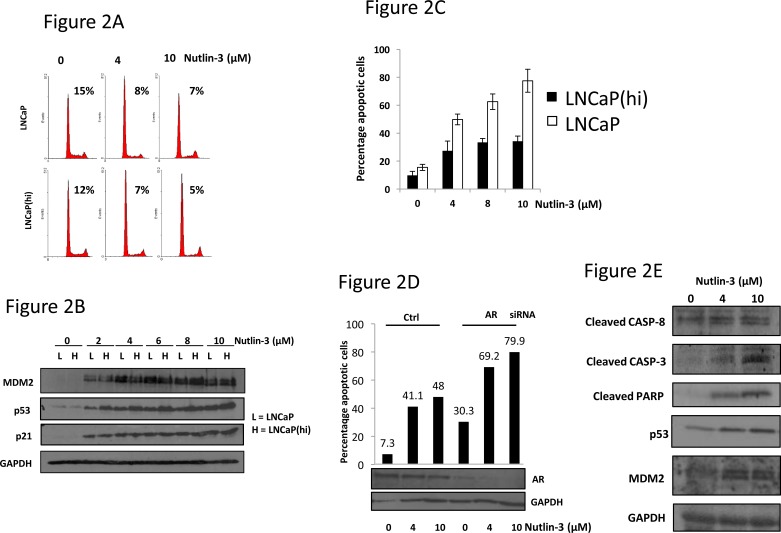
Nutlin-3 enhances apoptosis, but not cell cycle arrest, in a manner dependent upon AR status **A.** LNCaP or LNCaP(hi) cells were treated with Nutlin-3 for 24 hr prior to propidium iodide staining and flow cytometry. Percentages indicate proportion of gated cells in S-phase. **B.** Cells treated as in (A) were subject to immunoblotting with the indicated antibodies. **C.** Cells were treated with Nutlin-3 for 48 hr prior to active caspase-3 staining and analysis by flow cytometry. **D.** LNCaP cells were transfected with control (Ctrl) or AR-targeting siRNAs for 36 hr prior to treatment with Nutlin-3 for 48hr at the doses shown. Cells treated in parallel were used for immunoblotting as shown. **E.** LNCaP cells were treated with Nutlin-3 as indicated prior to immunoblotting as shown. Data are representative of a single experiment, error bars ±SD.

To ascertain the mechanism responsible for these changes in proliferation, we evaluated cell cycle and apoptosis profiles in the LNCaP cells and LNCaP(hi) cells. Application of 4-10μM Nutlin-3 to either cell line, for 24 hr, resulted in a reduction in the number of cells in S-phase to similar levels between the cell lines (Figure 2A). Additionally, immunoblotting for p53, p21 and MDM2 demonstrated similar inductions in response to Nutlin-3 (Figure 2B) demonstrating a conserved p53 response between the cell lines. Moreover, silencing AR did not lead to an additional reduction in the number of cells in S-phase upon treatment with Nutlin-3, in either cell line, compared to a non-silencing siRNA (Supplementary Figure S1). p53 silencing, on the other hand, increased the number of cells in S-phase in the presence of Nutlin-3. Overall, we conclude that the differences in proliferation observed between the cell lines upon Nutlin-3 exposure cannot be ascribed to cell-cycle control mechanisms and the cell cycle arrest exhibited in response to Nutlin-3, which is common to both cell lines, is p53-dependent rather than AR-dependent.

Upon studying apoptosis in LNCaP cells versus LNCaP(hi) cells, we noted a striking difference between the cell lines in their response to Nutlin-3 (Figure 2C and Supplementary Figure S2). Whereas LNCaP(hi) cells exhibited 34.2 % apoptosis in response to 10μM Nutlin-3 after 48 hr treatment, LNCaP cells exhibited approximately 77.5 % apoptosis. Additionally, siRNA-mediated AR silencing led to a large increase in the apoptotic population in LNCaP cells treated with Nutlin-3, compared to a non-silencing control siRNA (Figure 2D and Supplementary Figure S3). We also observed increases in cleaved CASPASE-3 and -8, cleaved PARP and p53 in LNCaP cells in response to Nutlin-3, in keeping with Nutlin-3 causing apoptosis (Figure 2E). Taking all observations together, we conclude that the correlation between AR dependency and Nutlin-3 sensitivity observed in the proliferation assays is due to changes in apoptosis rather than cell cycle arrest, but the p53 response is similar between the cells lines.

### Nutlin-3 enhances AR-MDM2 interaction, leading to AR ubiquitination

**Figure 3:**
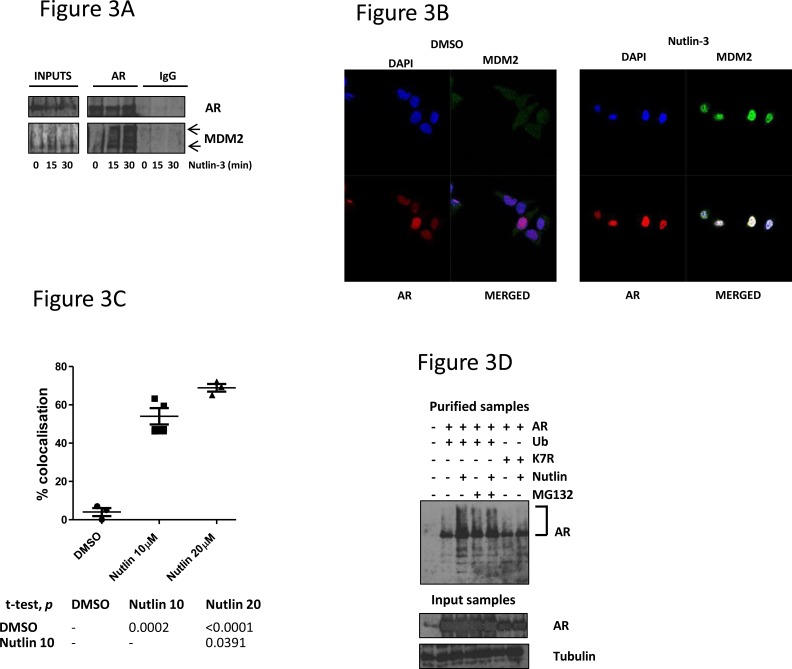
MDM2 interacts with AR in response to Nutlin-3 treatment, resulting in AR ubiquitination **A.** LNCaP cells were treated with 10μM Nutlin-3 prior to immunoprecipitation with either polyclonal AR or IgG control immunoglobulins before immunoblotting as indicated. Arrows indicate MDM2 species. **B.** LNCaP cells were treated with Nutlin-3 prior to dual immunofluorescence staining for MDM2 and AR as shown. **C.** Graph representing percentage of MDM2-AR co-localisation with associated statistical *p* value in table. **D.** 293T cells were transfected with the plasmids encoding CMV-driven AR, His-tagged ubiquitin or ubiquitin lysine mutant K7R prior to treatment with 10μM Nutlin-3 or 5μM MG132 for 24 hr. Purified nickel chromatography and input samples were subject to immunoblotting as indicated; AR-ubiquitin smears indicated by bracket.

In order to understand the mechanism through which Nutlin-3 impacts upon AR signalling, we examined the AR-MDM2 interaction. LNCaP cells were treated with Nutlin-3 for 15-30min prior to AR immunoprecipitation (Figure 3A). This short time period was used so that changes in MDM2 levels could not be ascribed as being responsible for any change in AR-MDM2 interactions. Whilst AR-MDM2 interactions were almost undetectable in material from untreated cells, the interaction could be readily detected in lysates from cells treated with Nutlin-3, without appreciable changes in the total cellular levels of MDM2 (Figure 3A). Immunoprecipitation with control IgG did not recover significant quantities of either AR or MDM2. We additionally tried to examine the p53-MDM2 complex by co-immunoprecipitation under the same conditions. Low basal levels of MDM2, combined with dynamic changes in p53 protein levels upon Nutlin-3 exposure made the results difficult to interpret (data not shown), but Nutlin-3 has previously been documented to inhibit the p53-MDM2 protein complex.

The subcellular localisation of AR and MDM2 was also examined in response to Nutlin-3 in LNCaP cells (Figure 3B). In untreated cells MDM2 did not appear to co-localise with AR in either the nucleus or the cytoplasm. Conversely, we estimate that more than 50% of AR co-localised with MDM2 in response to Nutlin-3, which was most apparent in the nucleus (Figure 3B and 3C). To determine the consequence of this AR-MDM2 interaction, we examined the ubiquitination status of AR in response to Nutlin-3 using nickel chromatography assays to recover His-tagged ubiquitin from transfected 293T cells (Figure 3D). At shorter exposures to Nutlin-3, such as 15-30min, ubiquitination of AR was difficult demonstrate (not shown). Additionally, in the absence of Nutlin-3 only negligible quantities of ubiquitinated AR were observed. However, after 24hr treatment with Nutlin-3 we observed an enrichment of high molecular weight AR species, presenting as a smear on immunoblotting (Figure 3D). Notably, treatment with the proteasome inhibitor MG132 generated a similar banding pattern, suggesting that these species represent ubiquitinated AR. This suggests that Nutlin-3 treatment promotes AR ubiquitination. Introduction of a vector carrying a mutant cDNA encoding a ubiquitin protein that is incapable of forming polyubiquitin chains (K7R), reduced the presence of these AR species back to baseline levels, reinforcing the notion that these high molecular weight species are indeed (poly)ubiquitinated AR (Figure 3D). Altogether, we suggest that Nutlin-3 treatment promotes AR-MDM2 interactions and subsequent AR ubiquitination.

### Nutlin-3 treatment reduces the expression of the AR-responsive c-FLIP gene

**Figure 4:**
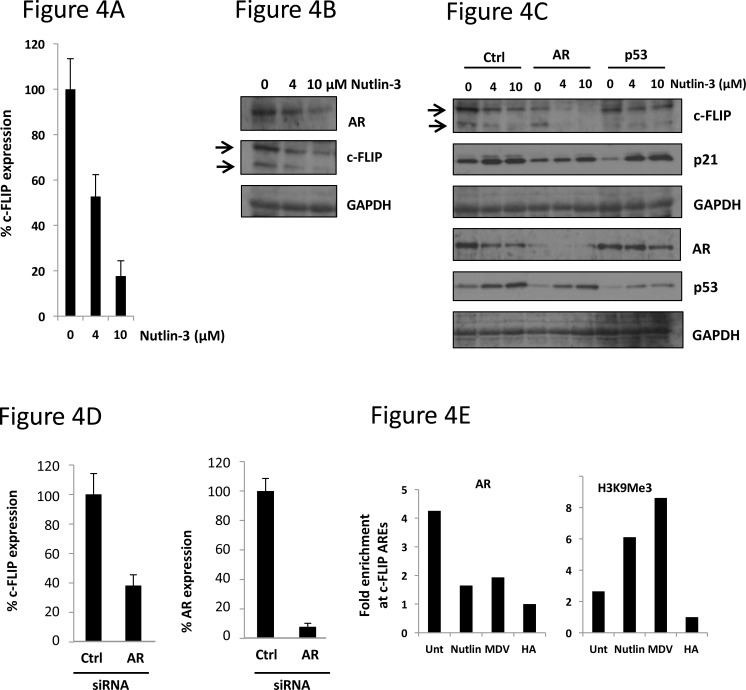
Nutlin-3 treatment results in loss of AR recruitment to the c-FLIP gene and downregulation of c-FLIP expression **A.** LNCaP cells were treated with Nutlin-3 for 48 hr prior to analysis of c-FLIP transcript levels, normalised to HPRT housekeeping gene expression. **B.** LNCaP cells treated as in A were subject to immunoblotting as indicated. **C.** LNCaP cells were transfected with the indicated siRNA then treated with Nutlin-3 for 48 hr prior to immunoblotting with indicated antibodies. **D.** LNCaP cells were transfected with the indicated siRNA prior to assessment of c-FLIP or AR transcript levels, as in (A). **E.** LNCaP cells were treated with either 10μM Nutlin-3 or 5μM MDV3100 prior to chromatin immunoprecipitation with either AR or histone H3 tri-methyl lysine 9 (H3K9Me3) immunoglobulins where shown. Quantities of c-FLIP ARE PCR products are corrected for input samples then expressed as fold change over HA immunoglobulin control sample, representative data.

Having demonstrated that exposure to Nutlin-3 leads to both apoptosis in cells dependent upon AR, we questioned what impact Nutlin-3 would have on AR-driven pro-survival genes. Previous studies have demonstrated that the androgen-responsive pro-survival *c-FLIP* gene is important in prostate cancer [11], making it a tractable target to study AR-mediated survival. To determine whether Nutlin-3 might influence *c-FLIP* expression, we studied both transcript and protein levels in LNCaP cells exposed to pro-apoptotic concentrations of Nutlin-3. After 48hr, exposure of LNCaP cells to Nutlin-3 reduced c-FLIP transcript levels by more than 80% (Figure 4A), and produced a large reduction in both detectable c-FLIP isoforms at the protein level (Figure 4B). Additionally, whilst silencing the AR in LNCaP cells led to an expected reduction in c-FLIP expression, treatment of these cells with Nutlin-3 produced further reductions in *c-FLIP* expression (Figure 4C and 4D).

In order to explain the observed decrease in expression of *c-FLIP*, we examined recruitment of AR to the *c-FLIP* gene by chromatin immunoprecipitation (ChIP) in LNCaP cells. Having shown a decrease in AR levels in response to Nutlin-3, we postulated that recruitment of AR to *cis*-acting androgen response elements (AREs) might also be impaired in this context. Specific oligonucleotides were used to amplify a region of the human *c-FLIP* promoter containing 4 characterised AREs upstream from the transcription start site. As shown in Figure 4E, treatment of cells with the AR antagonist MDV3100 resulted in a loss of AR recruitment, validating the ChIP method as a means to study AR recruitment to the c-FLIP gene. Interestingly, treatment with 10μM Nutlin-3 over 24 hours reduced AR recruitment to the *c-FLIP* promoter to levels detected with a non-specific control HA antibody (Figure 4E). An enrichment of the repressive histone H3 trimethyl lysine 9 (H3K9Me3) mark was also observed, further reinforcing the notion that exposure to Nutlin-3 leads to the formation of an inactive transcriptional state upon the c-FLIP gene, and a reduction in c-FLIP levels.

### Depletion of c-FLIP sensitises cells to Nutlin-3

**Figure 5:**
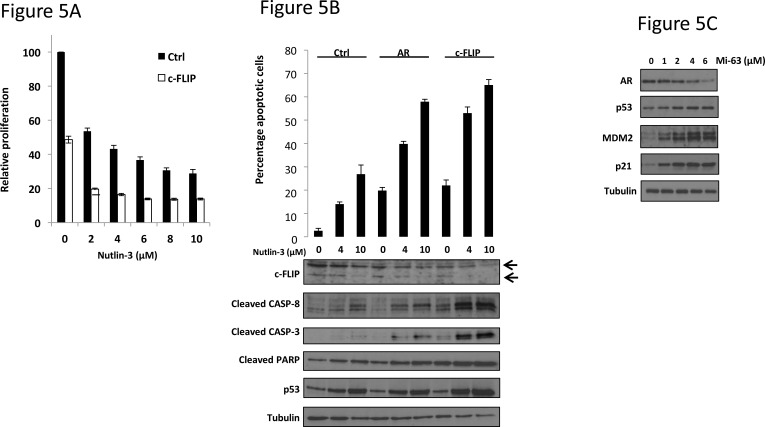
c-FLIP knockdown potentiates apoptosis in response to Nutlin-3 **A.** LNCaP cells were transfected with either control (Ctrl) or c-FLIP siRNA (*n* = 8) then treated with Nutlin-3 for 72 hr prior to WST-1 proliferation assay. **B.** LNCaP cells were transfected with the indicated siRNA prior to treatment with Nutlin-3 for 48 hr as shown. Cells were then subject to flow cytometry measurement of Annexin V or immunoblotting, as indicated. **C.** LNCaP cells were treated with Mi-63 for 36 hr prior to immunoblotting with the indicated immunoglobulins.

We hypothesized that if the decrease in c-FLIP levels observed upon Nutlin-3 treatment was in some way responsible for apoptosis, then silencing *c-FLIP* should enhance this effect yet further. Firstly, LNCaP cells were transfected with siRNA targeting c-FLIP prior to exposure to Nutlin-3. Quantitative PCR demonstrated that the c-FLIP siRNA was capable of decreasing c-FLIP transcript levels by approximately 80% (Supplementary Figure S4). Silencing c-FLIP alone led to a marked 50% reduction in proliferation compared to non-silencing control siRNA (Figure 5A). However, addition of Nutlin-3 even at doses as low as 2μM produced more than an 80% decrease in proliferation (Figure 5A).

We then examined apoptosis upon c-FLIP knockdown, in combination with Nutlin-3 (Figure 5B and Supplementary Figure S5). As expected, Nutlin-3 exposure led to an increase in the number of apoptotic cells, which was enhanced upon silencing AR. Notably, silencing c-FLIP sensitized cells to Nutlin-3 with a very large increase in apoptotic cell numbers over those cells transfected with control siRNA (Figure 5B). In order to demonstrate that the loss of c-FLIP in response to Nutlin-3, AR knockdown, or indeed c-FLIP knockdown has an impact on the expected downstream pro-apoptotic signalling pathway, we examined levels of cleaved CASPASE-3 and -8 in LNCaP cells. Exposure to Nutlin-3 led to an increase in the levels of both cleaved CASPASE-3 and -8, and this effect was notably enhanced upon silencing either AR or c-FLIP (Figure 5B). This data suggests that a decreased level of c-FLIP, in response to Nutlin-3 is at least partly responsible for the observed increase in apoptosis. The fact that AR silencing has a similar effect strongly suggests that Nutlin-3 does indeed impact on pro-survival AR signalling in human prostate cancer cells.

Finally, to demonstrate that downregulation of AR in response to p53-MDM2 inhibition is not specific to the Nutlin-3 compound we tested the structurally unrelated MDM2 antagonist Mi-63, previously shown to stabilise p53 and possess anti-proliferative properties in LNCaP cells [29]. Mi-63 treatment produced a dose-dependent increase in the expression of the known p53 targets *p21* and *MDM2* as expected, but also a reduction in AR protein levels. This demonstrates that other MDM2 inhibitors impinge upon AR signalling.

## DISCUSSION

Novel approaches to targeting AR are worthy of investigation and might be of clinical use in the treatment of prostate cancers that will otherwise overcome current anti-androgen treatments, leading to clinical relapse and the emergence of CRPC. Here we describe a mechanism by which the AR can be targeted for destruction in prostate cancer cells, in a manner that does not directly inhibit the AR itself or androgen metabolism, using MDM2 antagonists. Other work has demonstrated merit in an indirect approach to targeting AR [30], but ours is the first study, to our knowledge, showing that AR can be successfully down regulated by manipulating its natural pathway of destruction, to produce a reduction in pro-survival gene expression and consequent increase in apoptosis.

Our data show that the effects of Nutlin-3 on AR signalling and apoptosis occur in addition to the previously defined p53-mediated effects, and could be responsible for the marked sensitivity of LNCaP cells to Nutlin-3. Additionally, this sensitivity of LNCaP cells to Nutlin-3 demonstrates that *MDM2* gene amplification is not strictly required for the maximal efficacy of MDM2 antagonists. This may be clinically relevant, given that prostate cancers only rarely harbor MDM2 amplifications. Our data show that combined treatment with Nutlin-3 and AR antagonists results in widespread apoptosis. This suggests that an *in vivo* assessment of MDM2 antagonists in combination with conventional AR antagonists or newer agents such as MDV3100 is required, which should result in highly effective tumour regression and may delay or prevent the onset of castrate resistant disease.

There remain unanswered questions about the potential use of MDM2 antagonists in prostate cancer; the effects of Nutlin-3 on AR mutants and splice variants that arise in aggressive forms of the disease are not yet known and the mechanism by which MDM2 inhibitors other than Nutlin-3 could destabilise AR are currently unexplored. Additionally, the effects of Nutlin-3 and other MDM2 antagonists have not been thoroughly assessed in prostate cancer cell lines that express alternative forms of AR or mutant p53, such as VCaP or 22Rv1, although a direct comparison between such cells would prove difficult due to their rather divergent lineage. Importantly, although we suggest that downregulation of c-FLIP is important in the response to Nutlin-3 in AR-dependent cells, we have not yet obtained a global assessment of other androgen-regulated genes in this setting, which might shed light on alternative pro-survival mechanisms that could be altered in response to Nutlin-3, or explored as potential future prostate cancer treatments. Our attempts to perform a rescue experiment by transfection of c-FLIP prior to treatment with Nutlin-3 generated cytotoxicity too prohibitive to determine whether forced expression of c-FLIP alone could confer resistance to Nutlin-3. Nevertheless, the experiments in which we have inhibited AR either by siRNA or anti-androgens suggest that it is a target downstream of AR, such as c-FLIP which is important in maintaining cell survival and consequent disruption of this circuitry confers sensitivity to drug treatment.

Despite the above limitations, the majority of prostate tumors retain functional AR signalling and wild type p53, so we propose that prostate cancer could represent an ideal setting in which to make a clinical assessment of p53-MDM2 antagonists.

## MATERIALS AND METHODS

### Cell culture, transfection and immunofluorescence

Parental LNCaP cells were extensively cultured to > 50 passages to obtain high passage LNCaP(hi) cells. Casodex resistant LNCaP(CR) cells have been previously described [26]. Proliferation assays were performed using WST-1 reagent (Invitrogen) as described [22]. siRNA transfection was performed with RNAiMAX as per manufacturers recommendations with a final concentration of 25nM siRNA. For Immunofluorescence, cells were cultured for 72h on coverslips prior to treatment with either DMSO, 10-20μM Nutlin for 30 min. Cells were fixed with ice cold methanol, stained and imaged using Zeiss LSM 700 confocal microscope. Co-localisation of proteins was analysed using Zen2009 software to quantify average co-localisation of AR and MDM-2. All experiments have been performed 3 times.

### Flow cytometry

Cell cycle profiles were obtained at 24hr exposure to Nutlin-3, whilst cells remained viable, by permeablising in 1% Triton-X-100 in PBS followed by treatment with 100 μg/ml RNase and 500 μg/ml propidium iodide for 10 min. Cells were then washed in PBS prior to analysis.

Apoptosis was measured using either active caspase-3 (BD Pharmingen, cat 550480) or Annexin V / propidium iodide staining (BD Pharmingen, cat 556547) as described by manufacturer. No cells were gated out of analysis. In the case of caspase-3 a discrete population was observed and quantitated respective to the unstained population (see Figure S2). For Annexin V, all positively stained cells were counted (see Figure S3).

### Antibodies, reagents and oligonucleotides

Antibodies included MDM2 (Santa Cruz), p53 and p21 (Cell signalling technologies), AR (BD Biosciences), Caspase-3, 8 and PARP as described [27]. Nutlin-3, MG132 and siRNA were obtained from Sigma.

### Nickel chromatography, immunoprecipitation and chromatin immunoprecipitation

Ni-NTA agarose (Qiagen) was used to purify His-tagged ubiquitin modified proteins under reducing conditions as previously described [19]. Immunoprecipitation was performed after lysis in 50 mM Tris HCl, pH 7.4, with 150 mM NaCl, 1 mM EDTA, and 1% TRITON-X-100, supplemented with cOmplete protease inhibitors (Roche), using 1μg of AR or control immunoglobulin (Dako) bound to Protein G Sepharose (GE healthcare). Chromatin immunoprecipition was performed as described [28] and oligonucleotides sequences were used to amplify c-FLIP AREs; 5′ CGACGAGTCTCAACTAAAAGGGA 3′ and 5′ CGCTTCTCTCCTACACCTCCTC 3′.

## SUPPLEMENTARY MATERIALS FIGURES



## References

[1] Chen Y, Clegg NJ, Scher HI (2009). Anti-androgens and androgen-depleting therapies in prostate cancer: new agents for an established target. Lancet Oncol.

[2] Mitsiades N (2013). A road map to comprehensive androgen receptor axis targeting for castration-resistant prostate cancer. Cancer Res.

[3] Bishr M, Saad F (2013). Overview of the latest treatments for castration-resistant prostate cancer. Nat Rev Urol.

[4] Scher HI, Sawyers CL (2005). Biology of progressive, castration-resistant prostate cancer: directed therapies targeting the androgen-receptor signaling axis. J Clin Oncol.

[5] Attard G, Cooper CS, de Bono JS (2009). Steroid hormone receptors in prostate cancer: a hard habit to break?. Cancer Cell.

[6] Shen HC, Balk SP (2009). Development of androgen receptor antagonists with promising activity in castration-resistant prostate cancer. Cancer Cell.

[7] de Bono JS, Logothetis CJ, Molina A, Fizazi K, North S, Chu L, Chi KN, Jones RJ, Goodman OB, Saad F, Staffurth JN, Mainwaring P, Harland S (2011). Abiraterone and increased survival in metastatic prostate cancer. N Engl J Med.

[8] Irmler M, Thome M, Hahne M, Schneider P, Hofmann K, Steiner V, Bodmer JL, Schroter M, Burns K, Mattmann C, Rimoldi D, French LE, Tschopp J (1997). Inhibition of death receptor signals by cellular FLIP. Nature.

[9] Gao S, Lee P, Wang H, Gerald W, Adler M, Zhang L, Wang YF, Wang Z (2005). The androgen receptor directly targets the cellular Fas/FasL-associated death domain protein-like inhibitory protein gene to promote the androgen-independent growth of prostate cancer cells. Mol Endocrinol.

[10] Cornforth AN, Davis JS, Khanifar E, Nastiuk KL, Krolewski JJ (2008). FOXO3a mediates the androgen-dependent regulation of FLIP and contributes to TRAIL-induced apoptosis of LNCaP cells. Oncogene.

[11] McCourt C, Maxwell P, Mazzucchelli R, Montironi R, Scarpelli M, Salto-Tellez M, O'sullivan JM, Longley DB, Waugh DJ (2012). Elevation of c-FLIP in castrate-resistant prostate cancer antagonizes therapeutic response to androgen receptor-targeted therapy. Clin Cancer Res.

[12] Muller PA, Vousden KH (2013). p53 mutations in cancer. Nat Cell Biol.

[13] Taylor BS, Schultz N, Hieronymus H, Gopalan A, Xiao Y, Carver BS, Arora VK, Kaushik P, Cerami E, Reva B, Antipin Y, Mitsiades N, Landers T (2010). Integrative genomic profiling of human prostate cancer. Cancer Cell.

[14] Brooks JD, Bova GS, Ewing CM, Piantadosi S, Carter BS, Robinson JC, Epstein JI, Isaacs WB (1996). An uncertain role for p53 gene alterations in human prostate cancers. Cancer Res.

[15] Ittmann M, Wieczorek R, Heller P, Dave A, Provet J, Krolewski J (1994). Alterations in the p53 and MDM-2 genes are infrequent in clinically localized, stage B prostate adenocarcinomas. Am J Pathol.

[16] Brown CJ, Lain S, Verma CS, Fersht AR, Lane DP (2009). Awakening guardian angels: drugging the p53 pathway. Nat Rev Cancer.

[17] Vassilev LT, Vu BT, Graves B, Carvajal D, Podlaski F, Filipovic Z, Kong N, Kammlott U, Lukacs C, Klein C, Fotouhi N, Liu EA (2004). In vivo activation of the p53 pathway by small-molecule antagonists of MDM2. Science.

[18] Tovar C, Rosinski J, Filipovic Z, Higgins B, Kolinsky K, Hilton H, Zhao X, Vu BT, Qing W, Packman K, Myklebost O, Heimbrook DC, Vassilev LT (2006). Small-molecule MDM2 antagonists reveal aberrant p53 signaling in cancer: implications for therapy. Proc Natl Acad Sci U S A.

[19] Gaughan L, Logan IR, Neal DE, Robson CN (2005). Regulation of androgen receptor and histone deacetylase 1 by Mdm2-mediated ubiquitylation. Nucleic Acids Res.

[20] Lin HK, Wang L, Hu YC, Altuwaijri S, Chang C (2002). Phosphorylation-dependent ubiquitylation and degradation of androgen receptor by Akt require Mdm2 E3 ligase. EMBO J.

[21] Xia M, Knezevic D, Tovar C, Huang B, Heimbrook DC, Vassilev LT (2008). Elevated MDM2 boosts the apoptotic activity of p53-MDM2 binding inhibitors by facilitating MDMX degradation. Cell Cycle.

[22] Logan IR, McNeill HV, Cook S, Lu X, Lunec J, Robson CN (2007). Analysis of the MDM2 antagonist nutlin-3 in human prostate cancer cells. Prostate.

[23] Tovar C, Higgins B, Kolinsky K, Xia M, Packman K, Heimbrook DC, Vassilev LT (2011). MDM2 antagonists boost antitumor effect of androgen withdrawal: implications for therapy of prostate cancer. Mol Cancer.

[24] Scher HI, Beer TM, Higano CS, Anand A, Taplin ME, Efstathiou E, Rathkopf D, Shelkey J, Yu EY, Alumkal J, Hung D, Hirmand M, Seely L (2010). Antitumour activity of MDV3100 in castration-resistant prostate cancer: a phase 1-2 study. Lancet.

[25] Scher HI, Fizazi K, Saad F, Taplin ME, Sternberg CN, Miller K, de Wit R, Mulders P, Chi KN, Shore ND, Armstrong AJ, Flaig TW, Flechon A (2012). Increased survival with enzalutamide in prostate cancer after chemotherapy. N Engl J Med.

[26] Rigas AC, Robson CN, Curtin NJ (2007). Therapeutic potential of CDK inhibitor NU2058 in androgen-independent prostate cancer. Oncogene.

[27] Gamble LD, Kees UR, Tweddle DA, Lunec J (2012). MYCN sensitizes neuroblastoma to the MDM2-p53 antagonists Nutlin-3 and MI-63. Oncogene.

[28] Logan IR, McNeill HV, Cook S, Lu X, Meek DW, Fuller-Pace FV, Lunec J, Robson CN (2009). Heat shock factor-1 modulates p53 activity in the transcriptional response to DNA damage. Nucleic Acids Res.

[29] Ding K, Lu Y, Nikolovska-Coleska Z, Wang G, Qiu S, Shangary S, Gao W, Qin D, Stuckey J, Krajewski K, Roller PP, Wang S (2006). Structure-based design of spiro-oxindoles as potent, specific small-molecule inhibitors of the MDM2-p53 interaction. J Med Chem.

[30] Vanaja DK, Mitchell SH, Toft DO, Young CY (2002). Effect of geldanamycin on androgen receptor function and stability. Cell Stress Chaperones.

