# Supplementary material for: Nutlin-3 inhibits androgen receptor-driven c-FLIP expression, resulting in apoptosis of prostate cancer cells
Source: Oncotarget. 2016 Oct 9;7(46):74724–33. doi: 10.18632/oncotarget.12542 (PMC5342697; doi:10.18632/oncotarget.12542)
Supplement: Supplementary file 1 [file oncotarget-07-74724-s001.pdf]

Nutlin-3 inhibits androgen receptor-driven c-FLIP expression, resulting in apoptosis of prostate cancer cells

Supplementary Material

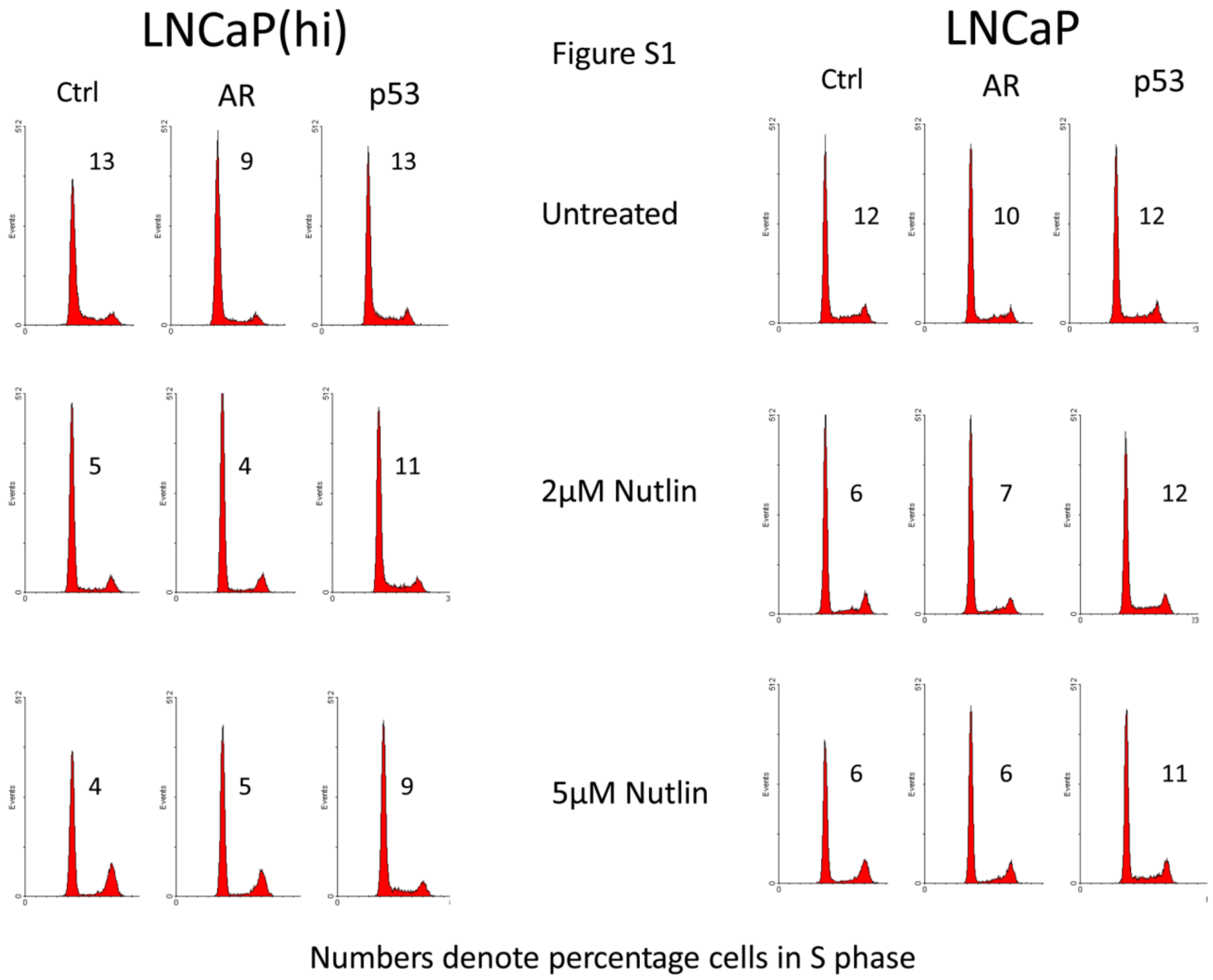

Figure S1. Representative cell cycle histogram profiles for LNCaP(hi) or LNCaP cells upon Control, AR or p53 siRNA transfection and treatment with Nutlin-3 for 24 hr. Numbers indicate % of cells in S-phase.

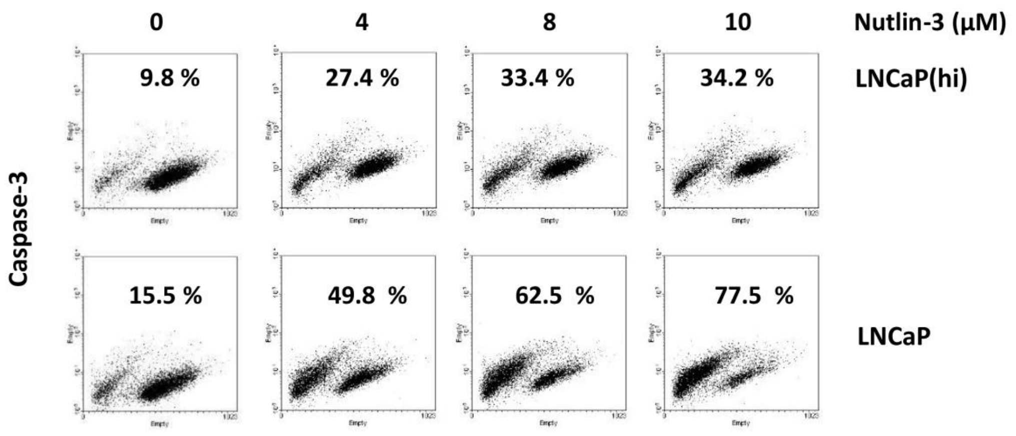

Figure S2. Representative scatter plots for Caspase-3 staining in LNCaP(hi) or LNCaP cells treated with Nutlin-3 for 24 hr. Numbers indicate % of apoptotic cells, capase-3 positive cells are the shifted population.

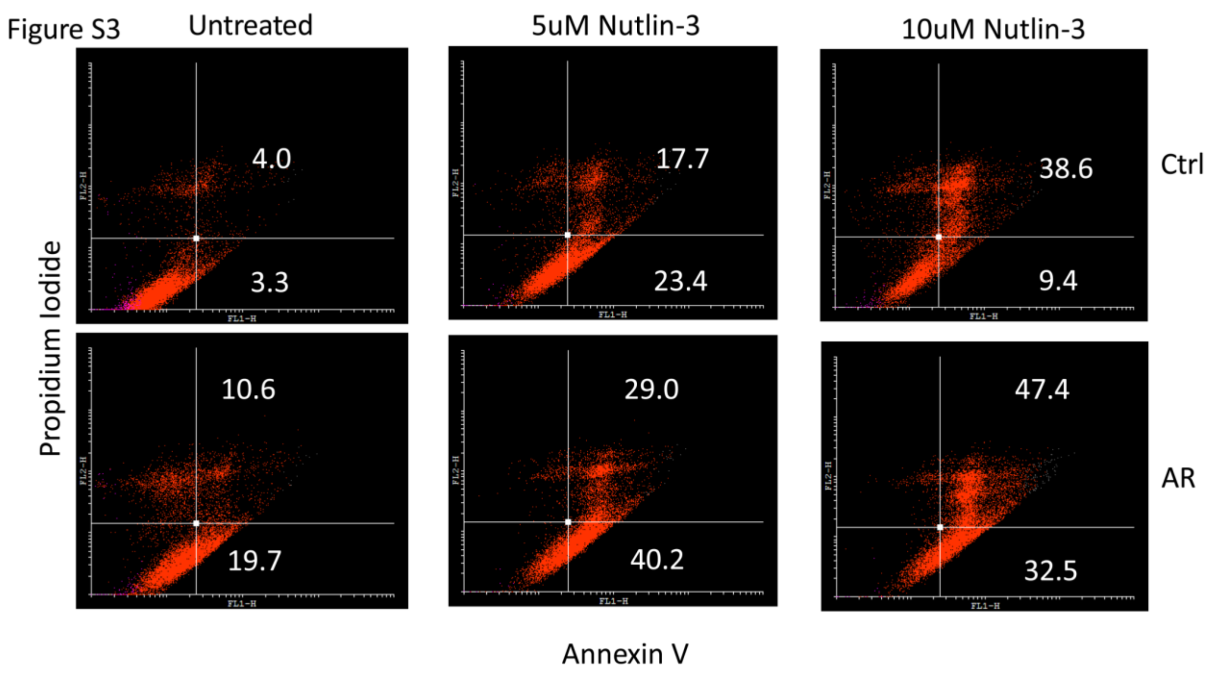

Figure S3. Representative scatter plots for Annexin V and propidium iodide staining in LNCaP cells after transfection of AR or p53 siRNA and treatment with Nutlin-3 for 48 hr. Numbers indicate % of apoptotic cells.

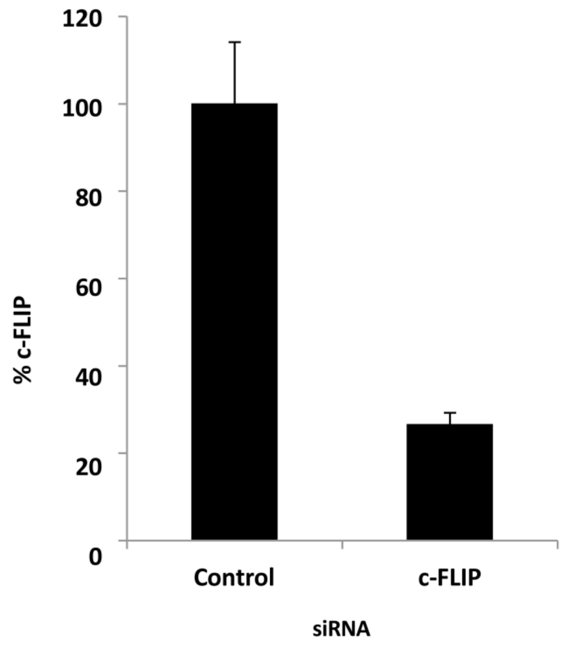

Figure S4. Representative demonstration of c-FLIP knockdown in LNCaP cells 36 hr post-transfection by quantitative PCR. Transcript levels were corrected to HPRT1 housekeeping gene.

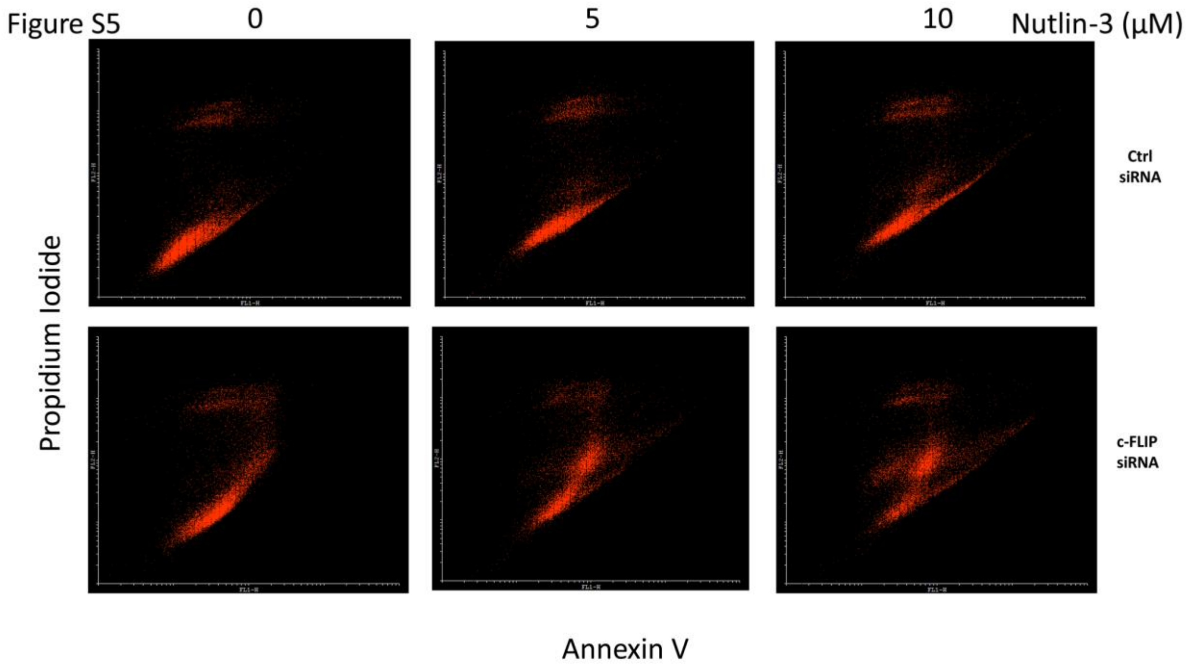

Figure S5. Representative scatter plots for Annexin V and propidium iodide staining in LNCaP cells after transfection of control (Ctrl) or c-FLIP siRNA then treated with Nutlin-3 as indicated for 48 hr.
